# Relapsed ocular squamous surface neoplasia treated with topical interferon alfa-2b

**Published:** 2020

**Authors:** Carlos Rocha-de-Lossada, Carmen Alba-Linero, Davide Borroni, Rahul Rachwani-Anil, Francisco Zamorano-Martín, Marina Rodríguez-Calvo-de-Mora

**Affiliations:** * Hospital Regional Universitario de Málaga, Servicio de Oftalmología, Spain; ** Department of Doctoral Studies, Royal Liverpool University Hospital, UK; Riga Stradins University, Riga, Latvia

**Keywords:** conjunctival intraepithelial neoplasia, ocular squamous surface neoplasia, limbal neoplasia, epidermoid carcinoma, impression cytology, Interferon α-2β

## Abstract

A 54-year-old patient presented with a large, fleshy conjunctival lesion in the right eye that had been previously surgically removed in another centre. Diagnosis of Conjunctival intraepithelial neoplasia (CIN) was confirmed by conjunctival impression cytology. The tumor was treated with topical IFN-α-2β (1,000,000 IU/ (ml) adjuvant to surgery for chemoreduction purposes. One month after treatment, the lesion showed almost complete involution, therefore the treatment was continued up to 6 months, and the lesion showed full regression. The patient is currently under periodical follow-up by conjunctival impression cytology.

We consider that topical IFN-α-2β can be used in CIN for chemoreduction and/ or curative purposes. We believe that conjunctival impression cytology is a useful and non-invasive method for diagnosis and follow-up in this condition.

## Introduction

Conjunctival-Corneal Intraepithelial Neoplasia (CCIN) is defined as a premalignant lesion that belongs to the group of the Ocular Surface Squamous Neoplasia (OSSN) [**1**], although there is a low probability of malignancy [**2**,**3**]. The yearly incidence is approximately 2 cases per 105 people. It usually presents in elder patients and the risk factors are mainly sun exposure, smoking, exposure to petroleum derivatives, human papillomavirus (HPV), human immunodeficiency virus (HIV), xeroderma pigmentosum, and stem cell therapy [**1**-**5**]. Differential diagnosis includes pterygium, corneal keratinization, conjunctival pannus, anterior basement membrane dystrophy, and malignant melanoma [**2**-**4**]. Clinical signs are typically a slowly progressive unilateral lesion, over elevated with a gelatinous, leukoplakia or papillomatous appearance that usually affects the limbal conjunctiva, usually located in the interpalpebral zone, and that extends over the corneal epithelium. Occasionally, nutrient vessels of the lesion can be observed, indicating a possible invasion of the basal membrane [**2**,**3**]. Classically, treatment has consisted in surgical excision and cryotherapy in the borders of the lesion [**1**-**3**]. However, there is a high recurrence rate as the border limits are imprecise [**1**,**3**]. In order to improve the surgical success rate, various coadjutant topical treatments such as 5-fluorouracil (5-FU), Mitomycin C (MMC) or Interferon-α-2β (INF-α-2β) have been employed [**1**-**3**]. 

INF-α-2β has been used as the first line treatment of OSSN, especially due to its low toxicity over the ocular surface and limbus [**1**-**3**]. 

We presented a case of a relapsed CCIN that, due to its large size, was treated with topical IFN-α-2β (1.000.000 UI/ ml) as a chemoreduction treatment prior to surgery. Nevertheless, the lesion experienced complete resolution with topical IFN-α-2β only; therefore, the patient is currently followed every 6 months in our centre, being subjected to periodical conjunctival impression cytology. 

## Case report

We presented the case of a 54-year-old patient with no relevant systemic personal history. In the ocular personal history, the patient had a previous surgery of a superior conjunctival lesion in the Right Eye (RE) in another centre, the result in the pathology report being negative for malignancy. Three years later, the patient was referred to our department due to a relapse of the conjunctival lesion.

**Fig. 1:**
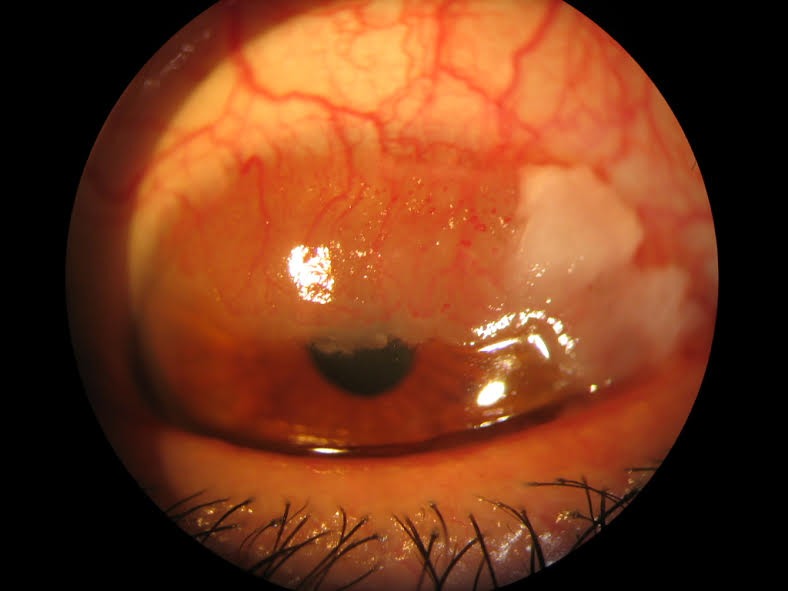
Prior to treatment with topical INFa-2b. The lesion affects approximately 180º of the superior conjunctiva and cornea, and it partially invades the pupil

On exploration, the RE presented a fleshy, leukoplakia, over elevated and vascularized lesion, extending approximately over the 180º superior limbus and superior conjunctiva (from IX to III) that invaded part of the pupil (**Fig. 1**). The Best Corrected Visual Acuity (BCVA) was 1.0 Snellen. Further anterior segment and fundus exploration were normal. Intraocular pressure was 20 mmHg in both eyes. 

As CCIN was suspected, conjunctival impression cytology samples were obtained, and resulted positive in the pathological reports, as squamous cells in different grades of malignancy were determined. In the context of a relapsed large size CCIN, chemoreduction topical IFN α-2β (1.000.000 UI/ ml) q.i.d. was administered prior to surgery.

**Fig. 2:**
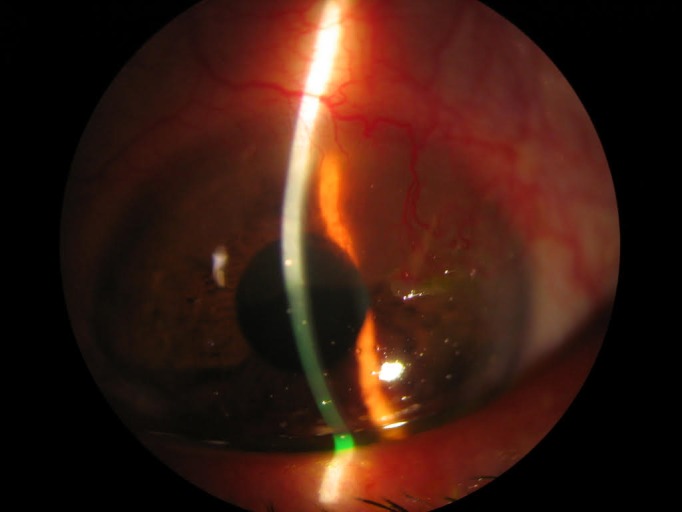
One month after topical treatment with INFa-2b. The lesion has significantly reduced in size and thickness, conjunctival leukoplakia has regressed, and pupillary axis was not affected

**Fig. 3:**
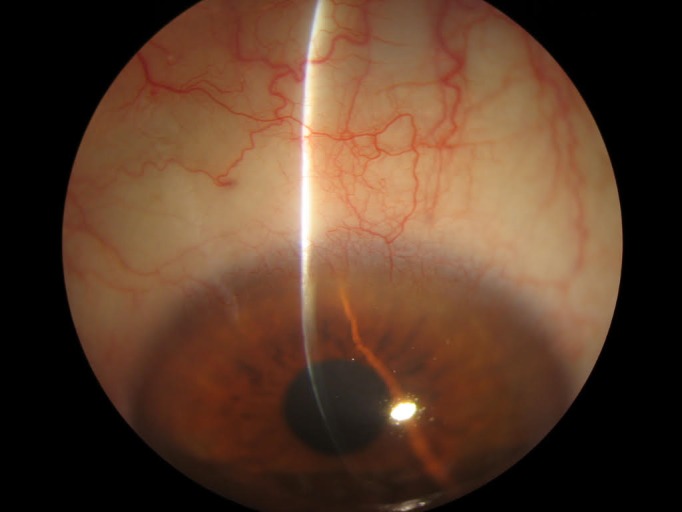
Six months after the initial treatment with topical INFa-2b. The lesion has disappeared

One month after treatment, the gelatinous and leukoplakia lesion diminished, showing flattening and regression of the vascularization (**Fig. 2**). Given the satisfactory response to IFN-α-2β, the treatment was continued up to six months of follow up, after which a complete remission of the lesion was noted (**Fig. 3**).

Currently, the patient is followed-up every six months in our centre, being subjected to conjunctival impression cytology. Until today, 36 months after the initial diagnosis, no relapse has been determined and the impression cytologies remained negative. 

## Discussion

CINN represent the most common neoplasia of the ocular surface [**2**,**3**,**6**] and include the first two phases of the spectrum of OSSN: conjunctival epithelial dysplasia, where dysplastic cells are confined to the basal layers of the epithelium; and carcinoma in situ, where dysplastic cells affect the whole thickness of the epithelium. The third stage of the disease corresponds to the squamous cell carcinoma, where malignant cells penetrate the basement membrane and invade the stroma. 

Although the certain diagnosis of OSSN is obtained by excisional biopsy and posterior anatomopathological examination of the specimen [**4**,**6**], clinical diagnosis is carried out by slit-lamp examination, reserving the biopsy for challenging cases [**2**,**3**,**7**]. 

Conjunctival impression cytology is a simple and useful technique for the diagnosis of conjunctival disorders such as limbal insufficiency, dry eye, and conjunctival neoplasia [**8**]. Its main advantage is the ability to obtain an epithelial specimen with minimum discomfort for the patient, precision in the collection of the specimen, and it allows a correct follow-up of a patient in order to detect relapse or progression after topical chemotherapy. CCIN cells can be detected by conjunctival impression, using predesigned paper strips, which are later treated with PAS dye, observing atypical pleomorphic cells with prominent nuclei [**6**]. Impression cytology has a high sensitivity in diagnosing CCIN [**7**]. The main limitation of this technique is that it does not distinguish a minimally invasive or advanced carcinoma in situ, as it only collects superficial cells.

Many diagnostic tests aid the clinical diagnosis. High definition OCT shows a thickened hyperreflective epithelial layer and an abrupt transition between normal and atypical epithelium. High-frequency ultrasonography displays lesions as a fusiform thickening with a hyperreflective surface, opposed to the hyporeflectivity of stromal tumors [**5**]. The later technique is also useful to evaluate a possible intraocular or orbital dissemination [**5**]. In vivo confocal microscopy has the power to obtain similar images to histological cuts of the ocular surface [**9**]. Nevertheless, collecting and interpreting the sample can be difficult. 

Classical treatment is surgical excision and cryotherapy on the surgical borders [**1**-**3**]. The most accepted surgical technique for localized lesions is the “no-touch” technique [**10**], which consists in the excision of the lesion avoiding any contact with it, ample margins (4mm), absolute alcohol instillation in the surgical bed, cryotherapy on the conjunctival margins and diathermic cauterization of the episcleral vessels. Given the inaccurate detection of the margins of the CCIN, recurrence rates represent from one third to half of the cases, depending on the series. Recurrence is more often in advanced stages and in multifocal lesions, hindering the complete excision and enhancing the incidence of tumoral recurrence [**1**-**3**].

In order to increase the success rates, various coadjutant treatments such as 5-FU, MMC or INF-α-2β, have been used [**1**-**3**]. IFN-α-2β has been used as both the main treatment, as well as a coadjutant, in CCIN, helping to avoid excessive scarring, damage of the limbal stem cells, and debulking the tumor prior to surgery [**1**-**3**]. Schechter reported a complete tumor remission in 96.4% of his series, after applying topical IFN-α-2β [**3**]. Karp also reported the effectiveness of subconjunctival IFN-α-2β in the treatment of OSSN [**1**]. The most common side effects of systemic treatment with IFN-α-2β include flu-like symptoms, headache, and asthenia [**3**]. However, these are uncommon to happen if IFN-α-2β is given topically [**3**]. Schechter reported foreign body sensation, follicular reaction and localized conjunctival hyperaemia as side effects after topical administration. 5-FU, all-trans retinoic acid, or MMC have also been reported as topical coadjutant treatments; however, these can lead to more severe side effects such as limbal ischaemia [**2**].

As our patient presented with an approximately 180º superior conjunctival-corneal lesion, surgical excision affected a significant portion of the limbus, consequently increasing the stakes in leading to a limbal stem cell deficiency. Moreover, as the case could probably be a relapsed tumor, we opted to carry out an initial conservative treatment with topical IFN-α-2β, with the intention to debulk the tumor size. Surprisingly, a complete remission of the lesion was observed six months after the treatment. 

Tumor recurrence can occur up to 10 years after the surgical excision [**2**,**3**], hence a yearly follow up of the patient is recommended [**2**]. Until today, 36 months after diagnosis, we did not observe any relapse signs in our patient who continues with periodic biannual revisions in our centre, being subjected to conjunctival impression cytology.

## Conclusions

Treatment with topical IFN-α-2β, either as an adjuvant chemoreduction prior to surgery, or as a unique therapy, is a valid option for CCIN, especially for relapsed cases or large size lesions, such as our case.

Diagnosis and follow-up of the CCIN can be safely, easily, and on an outpatient-basis carried out by means of conjunctival impression cytology. The latter has proved high sensitivity and a cost-effectiveness diagnostic tool. We consider that this test should be performed on a routine basis in case of suspicion of any ocular surface neoplasia.

**Conflict of interest**

The authors declare no conflict of interest.
